# Two-pore domain potassium channels enable action potential generation in the absence of voltage-gated potassium channels

**DOI:** 10.1007/s00424-014-1660-6

**Published:** 2014-12-09

**Authors:** Georgina MacKenzie, Nicholas P. Franks, Stephen G. Brickley

**Affiliations:** 1 Department of Neuroscience, Tufts University School of Medicine, 136 Harrison Avenue, Boston, MA 02111 USA; 2 Biophysics Section, Department of Life Sciences, Imperial College London, South Kensington Campus, London, UK

**Keywords:** Action potential, Potassium channels, Conductance simulation

## Abstract

In this study, we explored the possibility that two-pore domain potassium (K_2P_) channels are sufficient to support action potential (AP) generation in the absence of conventional voltage-gated potassium (K_V_) channels. Hodgkin–Huxley parameters were used to mimic the presence of voltage-gated sodium (Na_V_) channels in HEK-293 cells. Recombinant expression of either TREK-1 or TASK-3 channels was then used to generate a hyperpolarised resting membrane potential (RMP) leading to the characteristic non-linear current–voltage relationship expected of a K_2P_-mediated conductance. During conductance simulation experiments, both TASK-3 and TREK-1 channels were able to repolarise the membrane once AP threshold was reached, and at physiologically relevant current densities, this K_2P_-mediated conductance supported sustained AP firing. Moreover, the magnitude of the conductance correlated with the speed of the AP rise in a manner predicted from our computational studies. We discuss the physiological impact of axonal K_2P_ channels and speculate on the possible clinical relevance of K_2P_ channel modulation when considering the actions of general and local anaesthetics.

## Introduction

Introduction of the Hodgkin–Huxley model [25] of action potential (AP) generation has been followed by an impressive functional and molecular characterisation of the diverse array of voltage-dependent conductances responsible for AP generation in the peripheral and central nervous system [6]. In contrast, the passive membrane leak that is a component of all Hodgkin–Huxley-based AP models has received relatively little direct attention. However, in a number of cell types, the two-pore domain potassium (K_2P_) channel family has been identified as a key determinant of the membranes resting potassium permeability [17, 22, 36]. The increased potassium permeability imparted by K_2P_ channel expression leads to an increased input conductance and hyperpolarised resting membrane potential (RMP). In addition, the current–voltage relationship of the potassium leak conductance deviates significantly from a linear ohmic leak due to the asymmetrical distribution of potassium ions across the membrane [14, 21, 26]. The functional significance of this non-linear behaviour is not often considered. In cerebellar granule neurons (CGNs), this open rectification has been shown to influence the voltage-dependence of the input conductance [9, 10, 12], and genetic ablation of specific K_2P_ channels (namely TASK-3 or KNCK9) from CGNs lead to a striking phenotype. The APs generated in CGNs from TASK-3 knockout mice are significantly broader and smaller [9]. Similar phenotypes have also been observed after genetic ablation or inhibition or K_2P_ channels in other neuronal subtypes [23] and in cardiac tissue (for a recent review see [43]). Slowing of both the AP upstroke and the repolarisation phase can be explained by changes in the membrane time constant, and this largely overlooked functional consequence of K_2P_ channel expression could have wider implications for AP generation in the central and peripheral nervous system.

**Fig. 1:**
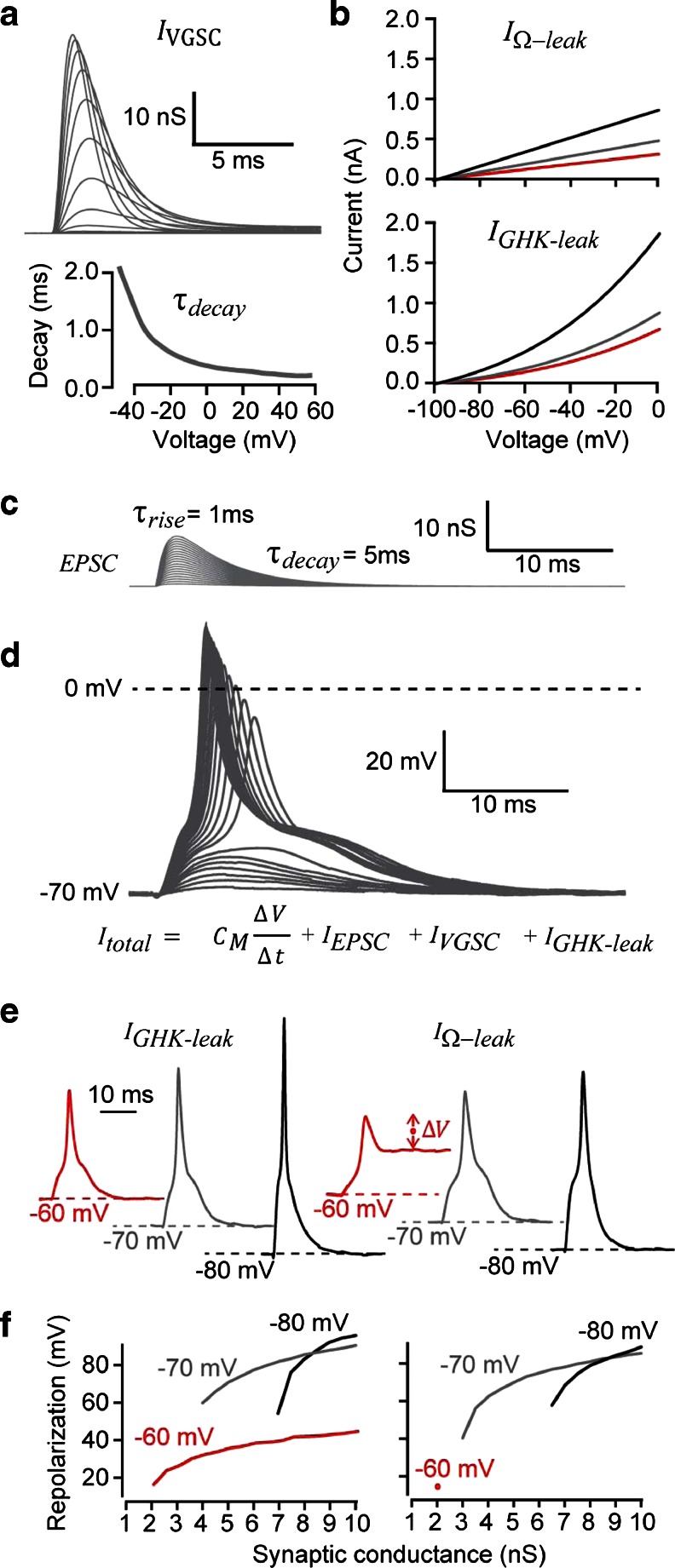
Simulations to compare the influence of linear versus non-linear leak conductance relationships on AP repolarisation. **a** Properties of the voltage-gated sodium current (*I*_VGSC_) used for simulation studies and dynamic-clamp experiments. The voltage and time-dependent conductance changes are plotted for simulations involving voltage steps from −40 to 0 mV in 5-mV increments. The voltage-dependence of ^τ^_decay_ for *I*_VGSC_ is also plotted. **b** The current–voltage relationships generated by the linear (*I*Ω_*-*leak_) and the non-linear potassium leak conductance (*I*_GHK-leak_) that generated RMPs of −60 mV (*red line*), −70 mV (*grey line*) and −80 mV (*black line*) are shown. The value of the linear leak conductance was 3.2 nS for −60 mV, 4.9 nS for −70 mV and 8.8 nS for −80 mV. The values of the GHK leak conductance at the minimum membrane potential (i.e. close to the RMP) was 0.65 nS at −60 mV, 0.85 nS at −70 mV and 1.8 nS at −80 mV **c** Superimposed synaptic currents (*I*_EPSC_) that were generated by double exponential functions with peak conductance values ranging from 0.5 to 10 nS. **d** The membrane voltage that resulted from summation of the currents generated by the conductance changes shown in **a**, **b** and **c** as well as the capacitive current generated by the voltage change. The superimposed voltage records were generated using the *I*_GHK-leak_ that resulted in an RMP of −70 mV. **e** Representative examples of voltage simulations comparing the linear (*I*Ω_*-*leak_) and the non-linear (*I*_GHK-leak_) leak conductance. APs are generated at all values of *I*_GHK-leak_ that fully repolarise back to the RMP. However, the lowest conductance value of *I*Ω_-leak_ that resulted in an RMP of −60 mV was insufficient to fully repolarise the RMP following stimulation by a 2-nS EPSC as shown by the trace indicated with a *red circle*. ***F***, Plots of AP repolarisation for all simulations indicating the linear (*I*Ω_*-leak*_) and non-linear (*I*_GHK-leak_) leak that was capable of repolarisation the AP. The only condition where this was not possible was at an *I*Ω_*-*leak_ resulting in an RMP of −60 mV. In this scenario, AP repolarisation was incomplete after the first EPSC of 2 nS, and no subsequent APs could be generated. This was in stark contrast to the *I*_GHK-leak_ that resulted in an RMP of −60 mV that was always sufficient to repolarise the AP

In this study, we tested the possibility that K_2P_ channels could both hyperpolarise the RMP and enable rapid AP repolarisation in the absence of K_V_ channels. The simulation data shown in Fig. 1a included voltage-gated sodium currents (*I*_VGSC_) that were based upon the Hodgkin–Huxley set of equations. As shown in Fig. 1b, we compared APs generated in the presence of either a linear leak (*I*_Ω − leak_) based upon a simple Ohmic behaviour or a non-linear leak (*I*_GHK ‐ leak_) based upon the Goldman–Hodgkin–Katz (GHK) model to mimic the non-linear leak that is known to be generated by K_2P_ channels (see Brickley et al. [9]). Both the *I*_Ω − leak_ and the *I*_GHK ‐ leak_ models reversed polarity at an equilibrium potential of -98 mV to mimic the presence of a potassium permeability, and the conductance magnitude was chosen to generate RMPs of −60, −70, and −80 mV. The synaptic-like waveforms (*I*_EPSC_) shown in Fig. 1c were constructed from double exponential functions and were used to depolarise the RMP to AP threshold. It is clear from the simulation data presented in Fig. 1d that the hyperpolarisation provided by a potassium leak conductance was sufficient to repolarise the AP in the absence of voltage-gated potassium channels. Moreover, as *I*_GHK ‐ leak_ increased so the AP duration got briefer due to the faster membrane time constant associated with the increased non-linear leak. As shown in Fig. 1e, f and a *I*_GHK ‐ leak_ that generates RMPs between −70 V and −80 mV is sufficient to repolarise the membrane potential during an AP, but an *I*_Ω − leak_ that generates an RMP of only −60 mV was not able to repolarise the AP. This computational data highlight the utility of a non-linear leak conductance of the type generated by K_2P_ channel expression.

Informed by these simulations, we went on to explore experimentally the ability of K_2P_ channels to repolarise the AP in the absence of K_V_ channels by using a dynamic-clamp procedure to mimic the presence of a sodium conductance in HEK-293 cells that were expressing the recombinant K_2P_ channels TREK-1 and TASK-3. In this way, we were able to demonstrate that K_2P_ channel expression alone was sufficient to generate APs, following Na_V_ activation/inactivation, in the absence of K_V_ channels. These experiments also demonstrated that, over a physiologically relevant range, AP shape could be altered by the potassium leak due to changes in the membrane time constant. The resistive shunt associated with a physiological K_2P_-mediated conductance did not negate AP generation but merely altered the current required to reach this threshold voltage. We went on to examine the consequence of enhancing the K_2P_-mediated leak conductance further with the volatile anaesthetic halothane. Once again AP attenuation was not observed at clinically relevant concentrations of halothane, but the enhancement of the K_2P_-mediated leak conductance resulted in a faster membrane time constant and therefore briefer APs. This hybrid simulation/expression approach demonstrates how AP properties could be influenced by K_2P_ channel expression in the central and peripheral nervous system.

## Methods

### Cell culture and transfection

Modified HEK-293 cells (tsA-201) were grown as monolayers in humidified (95 % O_2_, 5 % CO_2_) incubators at 37 °C. Upon reaching suitable confluency, cells were transferred to poly-d-lysine-coated glass cover slips for transfection using a transient calcium phosphate procedure with two separate plasmid DNA constructs encoding mouse TASK-3 (0.2 μg/μl of TASK-3 DNA; [1]), human TREK-1 (0.5 μg/μl of TREK-1 DNA) and Green Fluorescent Protein (0.5 μg/μl of GFP DNA). Newly transfected cells were transferred from the laminar flow hood to a 37 °C, 97%O_2_, and 3%CO_2_ incubator for 16–18 h. The transfection mixture was removed; the cells washed twice in phosphate buffered saline and fresh culture medium applied before plates were returned to the incubator for a further 24 h prior to electrophysiological recording.

### Electrophysiology

Whole-cell recordings were made using either an Axopatch 200B amplifier or a Multiclamp 700B (Molecular Devices; Foster City, CA) run via a National Instruments digitization board (PCI-6052E; National Instruments, Austin, Texas) linked to a dedicated PC. All experiments were performed at room temperature (22–23 °C), and the pipette solution contained (in mM) 120 KCH_3_SO_4_; 4 NaCl; 1 CaCl_2_; 1 MgCl_2_; 10 HEPES; 5 EGTA; 3 Mg-ATP; and 0.3 Na-GTP (adjusted to pH 7.3 with KOH). Data acquisition and analysis were performed using WINWCP (version 3.3.3) kindly provided by John Dempster (©John Dempster; University of Strathclyde, UK). Voltage recordings were passed through a 2-kHz, −3-dB, 8-pole Bessel filter before digitization at a rate of 20 kHz. The blocking action of tetraethylammonium (TEA) on the endogenous voltage-gated potassium conductance was calculated from the steady-state current measured during standard voltage-step protocols. The TEA inhibition curve was calculated using a modified Hill equation. The potassium conductance (*G*_K_) was calculated from the steady-state current (*I*_ss_) recorded at a holding potential (*V*_h_) of −60 mV using the potassium equilibrium potential (*E*_K_) to estimate the driving force according to the relationship *G*_K_ = *I*_ss_/(*V*_h_ − *E*_K_). The relationship between *G*_K_ and the RMP was adequately described by a single exponential function of the form *y* = A*exp^(−*x*/*τ*)^ + *y*_asymptote_. The dynamic-clamp was implemented in a Labview 7.2 (National Instruments) software environment following adaptation of the original G-clamp software developed by Paul Kullmann [33]. A dynamically variable current was injected using a patch-clamp amplifier (interfaced to a slave computer via a National Instruments PCI-6052E DAQ board, Austin, Texas). To make use of the computers’ full bandwidth, the slave computer was running embedded software controlled by a second master PC via an Ethernet connection. Dynamic-clamp simulation experiments were also implemented within Signal version 5 (Cambridge Electronic Design; Cambridge, UK) running a Multiclamp 700B (Molecular Devices; Foster City, CA) via a CED 1401 plus mk2 (Cambridge Electronic Design; Cambridge, UK).

### Simulation of ionic conductance changes

The presence of a voltage-gated sodium conductance was based upon modified Hodgkin–Huxley equations:

For the activation gate (*m*)

$$ {\alpha}_m = \frac{0.36\left(V+33\right)}{1-{e}^{\frac{\left(-V-33\right)}{3}}} $$

$$ {\beta}_m = \frac{-2\ \left(V+42\right)}{1-{e}^{\frac{\left(V+42\right)}{20}}} $$

$$ m=m+\left(\frac{dt}{10}\right)\left({\alpha}_m\ \hbox{--} m\left({\alpha}_m+{\beta}_m\right)\right) $$

For the inactivation gate (*h*)

$$ {\alpha}_h = \frac{-0.1\left(V+55\right)}{1-{e}^{-\frac{V+55}{6}}} $$

$$ {\beta}_h = \frac{0.45}{1+{e}^{\frac{\left(-V\right)}{10}}} $$

$$ h=h+\left(\frac{dt}{2}\right)\left({\alpha}_h-h\left({\alpha}_h+{\beta}_h\right)\right) $$

For the total current generated by the voltage-gated sodium current (*I*_*VGSC*_)

$$ {I}_{\mathrm{VGSC}}={\overline{g}}_{\mathrm{Na}}{m}^2h\left({E}_{\mathrm{rev}}-V\right) $$

where *V* is the membrane potential, *m* and *h* are the activation and inactivation variables, *E*_rev_ is the reversal potential (set at +55 mV) and ${\overline{g}}_{\mathrm{Na}}$ is the peak sodium conductance (set at 350 nS).

For the leak conductance, the constant field or GHK model provides us with a quantitative description of a non-linear IV relation of the type produced by a K_2P_ channel population where *z* is the valence of charge; *F*, Faraday’s constant (coulombs per mole); *T*, temperature (degrees Kelvin); *R*, gas constant (joules per mole-Kelvin); *[k*_in_ *]* and *[k*_out_ *]*, concentration of potassium ions inside and outside the cell; and ${\overline{g}}_{\mathrm{GHKleak}}$ is the slope conductance at *V* → ∞ [9].

$$ {I}_{\mathrm{GHKleak}}=V\frac{{\overline{g}}_{\mathrm{GHKleak}}}{\left[{k}_{\mathrm{in}}\right]}\left[\frac{\left\{\left[{k}_{\mathrm{in}}\right]-\left[{k}_{\mathrm{out}}\right]{e}^{\left(\frac{-VF}{RT}\right)}\right\}}{\left\{1-{e}^{\left(\frac{-VF}{RT}\right)}\right\}}\right] $$

The linear leak conductance was based upon a simple ohmic resistance of the form:

$$ {I}_{\Omega \mathrm{leak}}={\overline{g}}_{\Omega \mathrm{leak}}\left({E}_{\mathrm{rev}}-V\right) $$

where *V* is the membrane potential, *E*_rev_ is the reversal potential and ${\overline{g}}_{\Omega \mathrm{leak}}$ is the value of the conductance. An *E*_rev_ of −98 mV was chosen to match the reversal potential of the GHK leak.

Synaptic conductance waveforms were based upon the exponential difference synapse model of the form:

$$ {I}_{\mathrm{EPSC}}(t)={\overline{g}}_{\mathrm{EPSC}}f\left\{{ \exp}^{\left(\raisebox{1ex}{$-t$}\!\left/ \!\raisebox{-1ex}{${\tau}_{\mathrm{decay}}$}\right.\right)} - { \exp}^{\left(\raisebox{1ex}{$-t$}\!\left/ \!\raisebox{-1ex}{${\tau}_{\mathrm{rise}}$}\right.\right)}\right\} $$

where *t* is the start of the synaptic waveform, ${\overline{g}}_{\mathrm{EPSC}}$ is the peak synaptic conductance (varied between 0.5 and 10 nS), *f* is a normalisation factor to ensure that the peak conductance equals ${\overline{g}}_{\mathrm{EPSC}}$ and the rising phase is set by the smaller of the two time constants *τ*_rise_.

Reliable performance of the dynamic-clamp system requires minimal variation, or jitter, from cycle to cycle so that the requested arithmetical operations are achieved during each real-time loop. Operationally, the maximum jitter reported by the system when implementing this simple GHK-type leak was 2 ± 0.05 μs, slowing down to 11 ± 0.08 μs with the voltage-gated sodium conductance. The properties of *I*_VGSC_ were chosen to ensure that all calculations were performed well within the sample interval of 50 μs.

### Statistical analysis

All statistical tests were performed using STATISTICA 5.1 (StatSoft, Tulsa, OK) and considered significant at *P* < 0.05. A Shapiro-Wilk test was used to determine whether measures were normally distributed, and differences between groups were examined using the Student’s *t* test or the Mann–Whitney *U* test when distributions were not normal.

## Results

### Elimination of endogenous K_V_ channels from the expression system

**Fig. 2:**
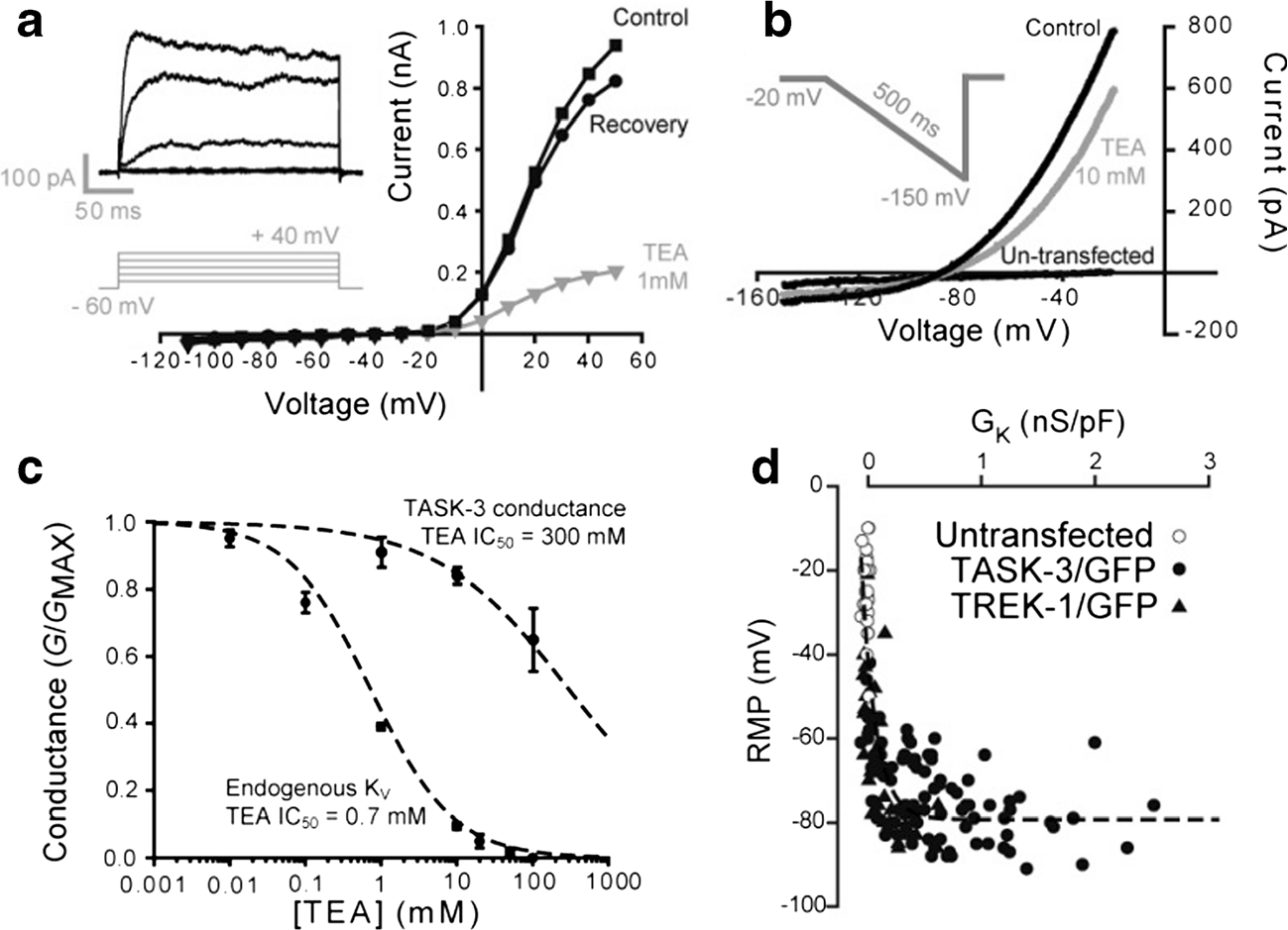
Removal of the endogenous voltage-gated potassium channels from the expression system. **a** Characterisation of conductances in HEK-293 cells illustrating the activation of an endogenous *K*_V_ near 0 mV. The *insert* shows a series of current records elicited by voltage steps from −100 to +40 mV. The current–voltage relationship of this *K*_V_ current has been plotted for recordings made in the presence (*grey triangles*) and absence (*black symbols*) of 1 mM TEA. **b** A voltage ramp from −20 to −150 mV was used to study the non-linear leak conductance present following TASK-3 and TREK-1 transfection. The current–voltage relationship of the resulting potassium leak conductance (*G*_K_) recorded from a TREK-1-transfected cell is shown in the presence (*grey trace*) and absence (*black trace*) of 10 mM TEA. For comparison, the current–voltage relationship for another un-transfected cell is also plotted to illustrate the small endogenous linear leak that is also present in these cells. Note the lack of any endogenous *K*_V_ activation over the voltage ranges examined in the absence of TEA. **c** Inhibition curves were constructed from data obtained following TEA block of the endogenous K_V_ and the TASK-3-mediated K_2P_ conductance. The data were well-described with a modified Hill equation (*dashed lines*) to give an estimate of the IC_50_ for this drug–receptor interaction. From these fits, it is clear that the endogenous *K*_V_ is sensitive to TEA with an IC_50_ of 0.7 mM, whereas the TASK-3 conductance is relatively insensitive to this blocker with an extrapolated IC_50_ of 300 mM. **d** In order to examine the relationship between *G*_K_ and the RMP, results were pooled from all cells transfected with either TASK-3 (*filled circles*) or TREK-1 (*filled triangles*), and for un-transfected cells (*open circles*). The data set was well described by a single exponential function (*dashed line*)

Before undertaking the dynamic-clamp experiments, we needed to ensure that endogenous K_V_ channels did not contribute to AP repolarisation. Consistent with the presence of an endogenous K_V_ channel population [47], there was a clear increase in membrane conductance (Fig. 2a) at command voltages more depolarised than −20 mV, resulting in a steady-state outward conductance of 0.20 ± 0.02 nS/pF (*n* = 5). The *K*_V_ blocker TEA significantly reduced the amplitude of this voltage-activated conductance with an IC_50_ of 0.7 mM (Fig. 2c). No transient A-type conductance [28] was apparent in any recorded cell (Fig. 2a) with or without a prepulse to −110 mV.

### TEA sensitivity of TASK-3 and TREK-1 channels

Figure 2b shows the IV curves generated from a GFP-positive cell in the presence and absence of 10 mM TEA, demonstrating the modest TEA sensitivity of K_2P_ channels. In these voltage-clamp experiments, the K_2P_-mediated conductance is recorded during a ramp protocol from −20 to −150 mV to avoid any contamination from the endogenous K_V_ conductance. TEA produced a consistent block of both the TASK-3- and TREK-1-mediated potassium leak conductance (*G*_K_). As shown in Fig. 2c, we observed a fully reversible 15.8 ± 2.5 % block of TASK-3 (*n* = 9), and a 25.2 ± 0.6 % block of the TREK-1 (*n* = 4) mediated conductance at a TEA concentration of 10 mM. As shown in Fig. 2c, the IC_50_ for the K_2P_-mediated conductance was extrapolated to be in the region of 300 mM. Therefore, all subsequent experiments were performed in the presence of 10 mM TEA, providing a system in which the biophysical and pharmacological properties of recombinant proteins could be studied in the absence of *K*_V_ currents.

### Influence of TASK-3 and TREK-1 channel expression on G_K_ and RMP

Figure 2b also compares the IV curves generated from GFP-positive and GFP-negative (un-transfected) cells, demonstrating the characteristic non-linear conductance that results from open rectification of K_2P_ channels. The non-linear leak conductance generated in TASK-3/GFP-expressing cells increases from 0.63 ± 0.01 nS/pF at −60 mV (*n* = 101) to 1.05 ± 0.01 nS/pF at −20 mV (*n* = 101). On average, TREK-1/GFP expression resulted in a smaller non-linear leak conductance that increases from 0.14 ± 0.01 nS/pF (*n* = 30) at −60 mV to 0.31 ± 0.01 nS/pF (*n* = 30) at −20 mV. In contrast, GFP-negative cells exhibited a small inward leak that was linear over the −60 to −20 mV range, with an average conductance of only −0.01 ± 0.0002 nS/pF (*n* = 82). The difference in potassium leak generated by TREK-1 and TASK-3 channel expression most likely reflects differences in channel density. These differences should influence the cells RMP. Indeed, TREK-1 transfected cells had average RMPs of −64.9 ± 0.7 mV (*n* = 31) compared with −73.0 ± 0.1 mV (*n* = 103), in TASK-3-expressing cells. Nevertheless, both of these RMPs were significantly more hyperpolarised than un-transfected cells (−24.6 ± 0.2 mV; *n* = 36). As shown in Fig. 2d, plotting the relationship between *G*_K_ and RMP for all GFP-negative, TASK-3/GFP- and TREK-1/GFP-expressing cells (*n* = 212) results in a distribution that is well described by a first-order exponential function reaching asymptote (−79.3 mV) at around 0.5 nS/pF.

#### The minimum requirements for AP propagation

**Fig. 3:**
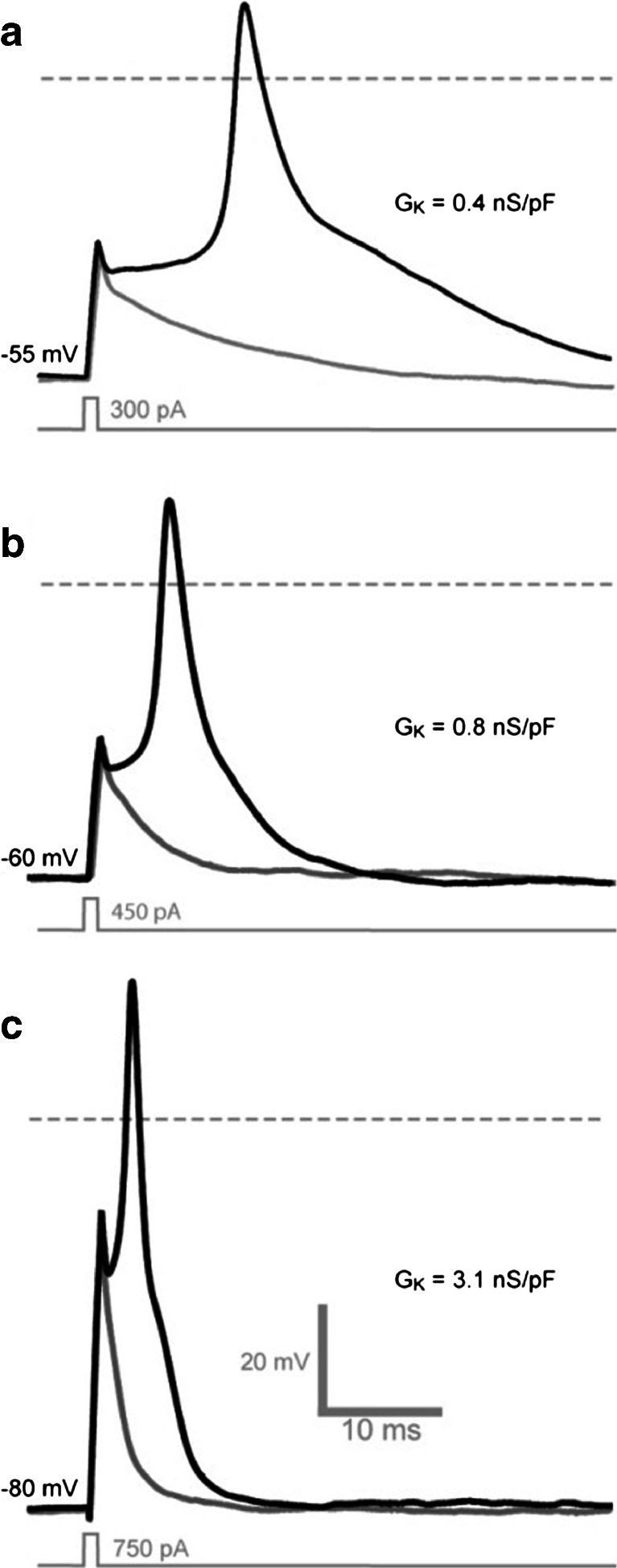
Two-pore domain potassium channel expression is sufficient to repolarise the AP. **a**, **b** and **c** Single AP generated in response to a brief 1-ms current injection in three individual cells expressing TREK-1 channels. The *grey dashed line* illustrates the 0 mV level. The magnitude of *G*_K_ was smallest in **a** (0.4 nS/pF) and largest in **c** (3.1 nS/pF). The magnitude of *G*_K_ correlates with the RMP of the HEK cell, the current required to reach AP threshold, and the duration of the AP. The sub-threshold voltage response is also shown for each cell (*grey line*) to further demonstrate the faster membrane time constant that is apparent at larger values of *G*_K_

In order to mimic the presence of Na_V_ channels in these cells, transient conductance changes of the type shown in Fig. 1 were mimicked by a simple set of Hodgkin–Huxley equations (see “Methods”). AP generation was not possible in GFP-negative cells. However, implementing a virtual sodium conductance in TREK-1-expressing cells resulted in robust APs with fast rise and decay and none after hyperpolarisation. This was true of all TREK-1- and TASK-3-expressing cells examined (*n* = 20). Although a K_2P_-mediated potassium leak conductance was clearly sufficient to repolarise the membrane, it was also clear that the AP width and height was variable from cell to cell. This variability in AP shape correlated with the magnitude of *G*_K_, as did the RMP and the current required to achieve AP threshold. Comparing the three examples shown in Fig. 3, it is clear that a larger *G*_K_ results in a faster membrane time constant and a correspondingly briefer AP. The faster membrane time constant is apparent in the subthreshold membrane response shown for each example. These data demonstrate how K_2P_ channel expression will be sufficient to repolarise the membrane following the activation/inactivation of Na_V_ channels leading to rapid Na_V_ deactivation in the absence of functional *K*_V_ channels. Also note that AP generated in the absence of Kv channels lack membrane after hyperpolarisation which is seen when *K*_V_ channels are also present. Membrane after hyperpolarisation facilitates the recovery of Na_V_ channels from inactivation and therefore aids high frequency firing.

#### Ability of K_2P_ channels to support sustained AP firing

**Fig. 4:**
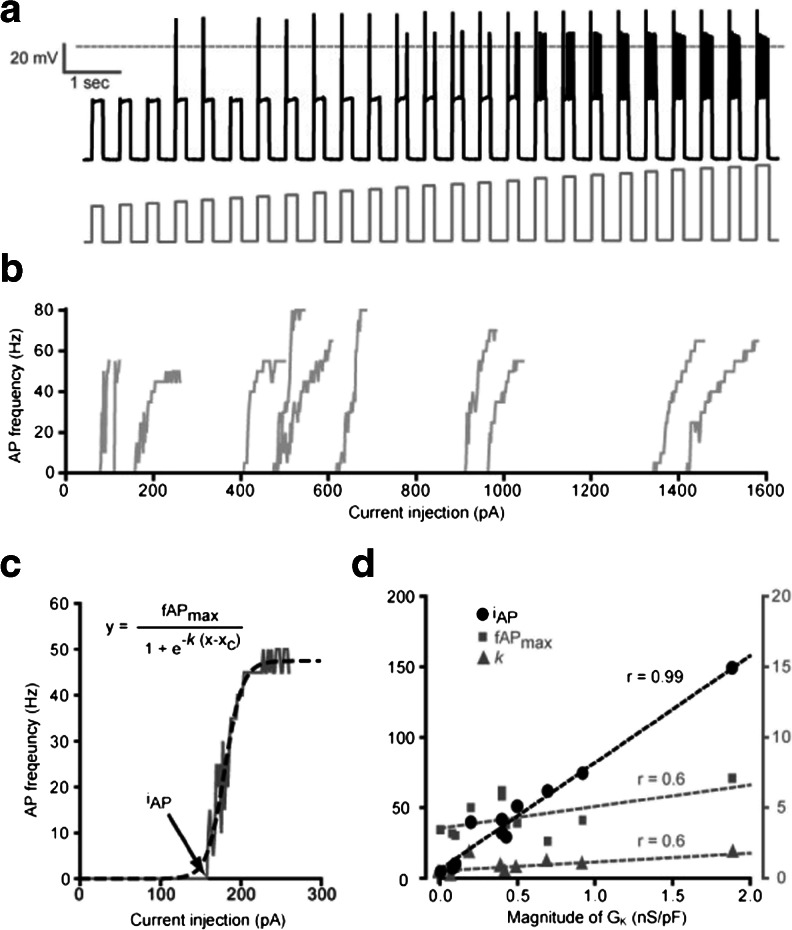
K_2P_ channel expression alters the current required to reach AP threshold. **a** Continuous voltage recording (*black trace*) obtained during a conductance simulation experiment from a HEK cell expressing TASK-3 channels. A series of long-duration 300-ms depolarising current pulses (*grey trace*) were applied at increasing current intensities. The relationship between the magnitude of the injected current and the AP frequency for all cells examined in this way is plotted in panel **b**. Analysis of a single cells AP frequency response to injected current is shown in panel **c**. This relationship is well described by a simple Boltzmann-type function (*dashed line*). From this analysis, we can estimate the current required to reach AP threshold (*i*_AP_), the slope of the relationship (*k*) and the maximum AP frequency (fAP_max_). **d** The results of fitting linear regressions to the relationship between *G*_K_ and the three variables (*i*_AP_, *k*, and fAP_max_) extracted from the data shown in **b**. It is clear that the only correlation is between the current required to reach AP threshold (*i*_AP_) and the magnitude of the potassium leak (*G*_K_)

Next, we examined whether the presence of K_2P_ channels enables sustained AP firing in response to a tonic depolarising stimulus. At low levels of K_2P_ channel expression, the ability to generate a single AP in response to a brief current pulse did not translate into continuous AP firing during sustained depolarisation. The top panel in Fig. 3 illustrates a cell that was not able to support continuous AP firing during longer current pulses, although the potassium leak conductance was able to repolarise the membrane during a single AP. In this cell, activation of the virtual sodium conductance at around −40 mV results in the initial AP upstroke, but the membrane was then maintained at around −20 mV throughout the depolarising current pulse (data not shown). High-frequency AP firing was apparent during sustained depolarisation only in those cells that exhibited larger *G*_K_ values. For example, the middle trace in Fig. 3 is taken from a cell with a *G*_K_ of 0.8 nS/pF, and this cell did support high-frequency sustained firing during longer depolarising current pulses. Differences in *G*_K_ also explained why in TASK-3-expressing cells the current required to reach AP threshold (*I*_AP_) for a 300-ms current pulse was 45.4 ± 12.4 pA/pF (*n* = 11) compared with 25.6 ± 10.3 pA/pF (*n* = 6) in TREK-1-expressing cells. Figure 4d also shows that, between cells, the variability in *I*_AP_ was clearly correlated with differences in *G*_K_, but, once AP threshold was reached, the slope of the change in AP firing rate together with the maximum AP frequency did not correlate with *G*_K_.

**Fig. 5:**
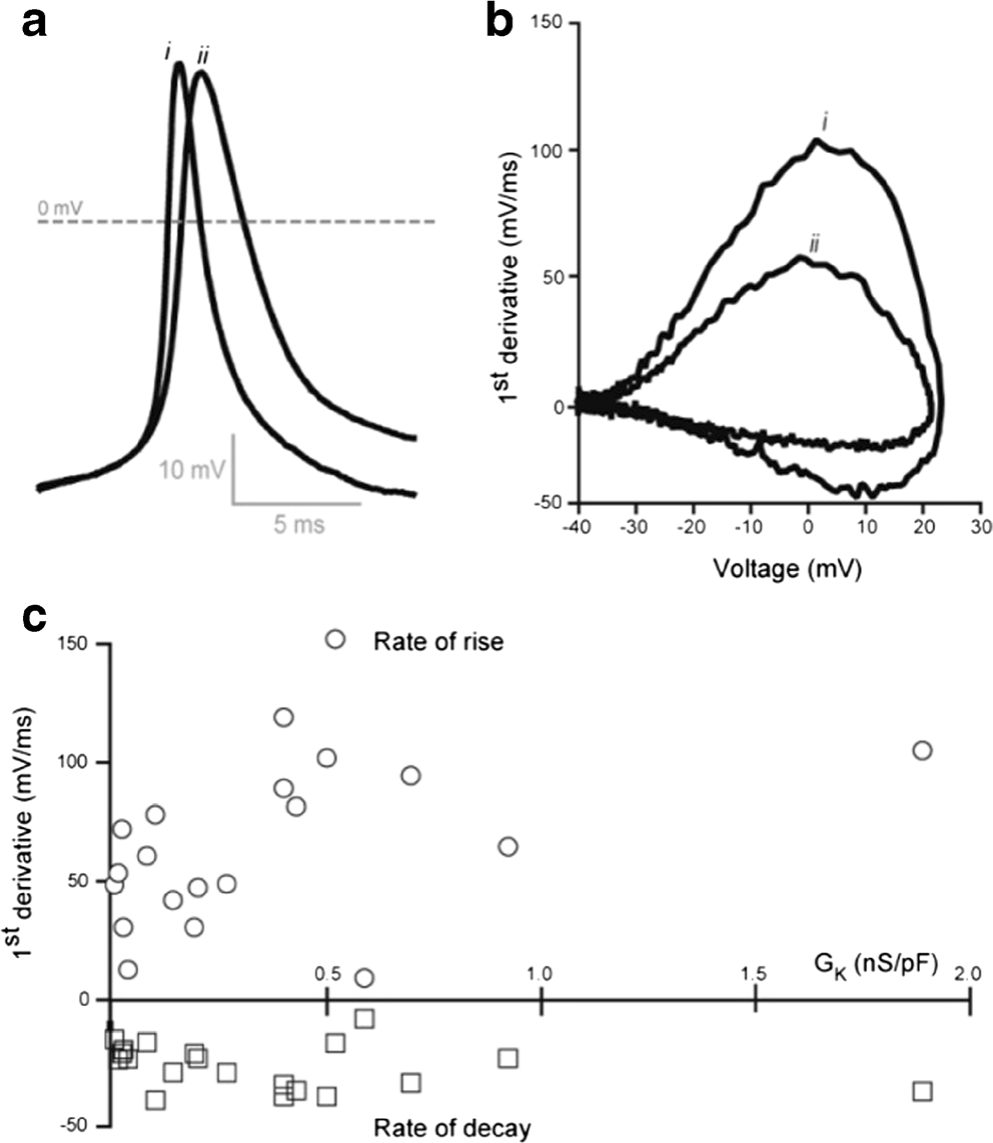
Relationship between the magnitude of the K_2P_-mediated conductance and the AP rise and decay. **a** Two threshold APs from separate cells are superimposed to illustrate the difference in AP rise and decay. The first AP (*i*) comes from a cell with a *G*_K_ of 0.1 nS/pF whereas the second AP (*ii*) comes from a cell with a *G*_K_ of 0.5 nS/pF. **b** Phase plane plots of the two APs shown in **a**. The maximum AP rise and decay was calculated from these plots. **c** Plot of the maximum AP rise (*open circles*) and decay (*open squares*) for all threshold APs elicited during conductance simulation procedure in relation to the *G*_K_ for each cell examined

Overall, as well as demonstrating the ability of the K_2P_-mediated conductance to support AP firing, these conductance simulation experiments demonstrate how a larger *G*_K_ results in a faster membrane time constant and therefore a briefer AP. The influence of *G*_K_ on the AP rise, peak and decay for all cells examined during the conductance simulation protocol is shown in Fig. 5. There is no obvious relationship between the magnitude of *G*_K_ and the AP peak amplitude, but the rate of AP rise clearly increases from 0 to 0.5 nS/pF (*n* = 15). The slower rate of AP decay is less obviously affected by alterations in *G*_K_. During these particular conductance simulation experiments, only five cells generated K_2P_ leaks exceeding 0.5 nS/pF, and we did not often encounter cells with G_K_ greater than 2 nS/pF. Therefore, it is difficult to comment on the relationship between *G*_K_ and AP properties at higher levels of K_2P_ expression, but the rate of AP rise peaks in the region of 0.5 nS/pF. It is also clear from these data that, over the range of *G*_K_ examined, AP peak and decay were not altered.

#### Effect of a volatile anaesthetic on cell excitability

**Fig. 6:**
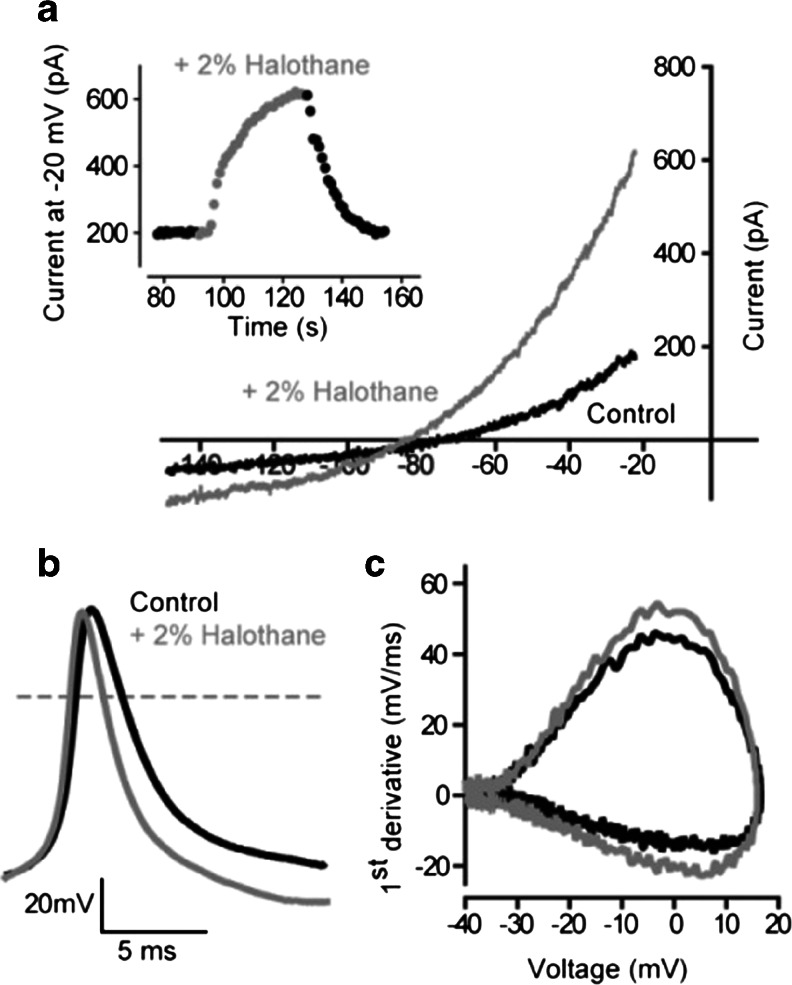
Actions of halothane on K_2P_ channels and the consequence of this enhancement for AP properties. **a** Results from a voltage-clamp experiment illustrating the current–voltage relationship for a TASK-3-expressing cell during a standard ramp protocol in the presence (*grey trace*) and absence (*black trace*) of 2 % halothane. The *insert* shows the time course of the halothane enhancement for this cell calculated at a command potential of −20 mV. **b** A single threshold AP elicited from a different TASK-3-expressing cell in the presence (*grey trace*) and absence (*black trace*) of 2 % halothane. The *dashed line* illustrates the 0 mV level. **c** Phase plane plots for the APs shown in **b** illustrating the modest speeding of the AP rise and decay in the presence (*grey trace*) of 2 % halothane

The relationship between *G*_K_ and AP properties was also examined by taking advantage of the enhancement of K_2P_ channel activity with the volatile anaesthetic halothane [37]. As shown in Fig. 6a, voltage-clamp experiments confirmed that bath application of 2 % halothane resulted in a significant 74 ± 36 % (*n* = 5) increase in the magnitude of *G*_K_. Although the halothane-induced increase in *G*_K_ was modest at this relatively low concentration, it was consistent, increasing from an average value of 0.6 ± 0.3 to 0.7 ± 0.1 nS/pF. In a separate series of conductance simulation experiments (*n* = 7), the result of this increased *G*_K_ was assessed on AP properties. No significant change in the AP peak amplitude (21.9 ± 2.7 mV versus 22.6 ± 3.1 mV) was observed, but, as shown in Fig. 6b, the rate of AP rise and decay were both significantly increased. The average rate of AP rise was increased by 13.2 ± 7.0 % (*n* = 7), from an average value of 83.8 ± 14.0 to 93.6 ± 15.0 mV/ms in the presence of 2 % halothane. The rate of AP decay was increased by a similar degree (16.0 ± 7.9 %), from an average value −28.6 ± 4.3 to −31.6 ± 3.6 mV/ms. It is interesting to note that there was no significant change in the RMP during halothane application, reflecting the relationship between *G*_K_ and RMP that has been illustrated in Fig. 2d. It can be seen from this relationship that, in situations where the cells RMP is dominated by the membranes potassium permeability, asymptote is reached at *G*_K_ values close to 0.5 nS/pF, and any further increase in *G*_K_ will not impact on the RMP. However, one other functional consequence of increasing *G*_K_ with halothane was reflected in the current required to reach AP threshold that increased in all cells examined from an average value of 31.7 ± 9.4 to 36.9 ± 9.4 pA/pF (*n* = 6).

## Discussion

Recombinant expression procedures were used to examine the biophysical impact of K_2P_ channel expression on AP generation by implementing a virtual voltage-gated sodium conductance in the HEK cells. This application of the conductance simulation approach clearly demonstrates that *K*_V_ channels are not an absolute requirement for AP generation, and the voltage-independent potassium leak conductance generated by K_2P_ channels is sufficient to repolarise the AP. In light of this observation, we discuss the role of axonal K_2P_ channels in AP generation and consider the impact of K_2P_ channel modulation during anaesthesia.

The endogenous *K*_V_ conductance present in these cells is small compared with the recombinant K_2P_ conductance, but it was still necessary to pharmacologically remove this confounding influence during the conductance simulation experiments. Performing experiments in the presence of 10 mM TEA reduced the magnitude of the recombinant K_2P_-mediated conductance, but the remaining non-linear leak conductance was clearly sufficient to enable AP generation in a manner predicted from our simulation studies. The magnitude of the *G*_K_ produced by our expression system was never sufficient to generate a resistive shunt capable of complete AP attenuation. This could well reflect an upper limit for the expression of K_2P_ channels in biological membranes. Nevertheless, when considering the entire dataset, it is clear that a larger *G*_K_ results in briefer APs due to the generation of a faster membrane time constant. It is worth noting that the magnitude of *G*_K_ observed in this expression system correlates well with the values obtained in native neurones. For example, in adult CGNs, the standing outward potassium conductance is 0.5 nS/pF in wild-type mice and is reduced to 0.3 nS/pF in the TASK-3 KO mice [9]. In addition to broadening the AP, this reduction in the TASK-mediated conductance depolarises the RMP and ultimately leads to a failure to sustain AP firing during continuous excitatory drive as AP accommodation becomes more likely as the underlying Na_V_ channels experience significant steady-state inactivation at depolarised potentials. Obviously, the impact of K_2P_ channels on these various aspects of neuronal excitability may not be so pronounced in all cell types as the relative contribution of this potassium leak conductance will vary for different cell-types. Nevertheless, it is worth considering that any neuromodulation that results in a reduced K_2P_-mediated conductance, for example, activation of G-protein coupled receptors, will influence more than just membrane hyperpolarisation.

### Physiological implications of K_2P_ channel expression in axons

Recordings from mammalian myelinated axons are characterised by a conspicuous absence of *K*_V_ currents [11, 13, 27, 31]. It is now apparent that *K*_V_1-type channels are restricted to the juxtaparanodal region of mammalian axons [3, 39–42, 48, 49] and are effectively hidden under the myelin. The functional significance of juxtaparanodal *K*_V_ channels in mammalian axons is therefore unclear as they do not directly contribute to AP propagation [50]. To account for a lack of functional *K*_V_ channels, AP propagation in mammalian myelinated axons has been explained in terms of rapid sodium channel activation/inactivation in combination with a sizable leak conductance [24, 30]. Indeed, both the Chiu-Ritchie-Staff-Stagg model [13] and the Schwarz-Eikhof model [44] rely on a leak conductance to repolarise the AP in the absence of a *K*_V_-like conductance. Although a potassium leak conductance is evident in recordings from myelinated axons [32], the origin of the nodal leak conductance is still not certain, and its contribution to AP generation has not been critically evaluated. Indeed, it has been suggested that the voltage-independent leak may not reflect the presence of a nodal potassium channel but could involve an extracellular pathway beneath the myelin that connects the nodal and internodal regions [5, 7]. However, this hypothesis is partly based upon the unusual biophysical and pharmacological properties of this leak conductance, namely its voltage-independence and TEA insensitivity. Voltage-independence and TEA insensitivity are now known to be defining features of K_2P_ channels [17, 22, 30, 36]. It has recently been shown that KCNQ2/3 channels are localized to mammalian nodes where they contribute to the maintenance of the RMP [3, 15, 45]. However, due to their slow, voltage-dependent activation kinetics and TEA sensitivity [29], it is unlikely that they are the sole contributor to the potassium leak conductance or to the repolarisating phase of APs at the node. Indeed, blockade of KCNQ currents reveals a substantial leak component and does not prevent the repolarisation of the membrane following an AP [15, 45]. The suggestion that, in combination with Na_V_ channels, K_2P_ channels could provide the potassium leak necessary to generate APs in the absence of K_V_1channels is therefore intriguing [9, 30], but future studies are needed to directly examine the contributions made by K_2P_ channels to the potassium permeability of mammalian myelinated axons.

### Clinical implications of K_2P_ channel modulators

Anaesthetic mechanisms are often considered to involve interactions with a diverse array of receptor types, but it is also possible that the actions of specific anaesthetics could be explained by an interaction with a limited number of sites [18, 20]. Volatile anaesthetics, like halothane, are known to potently enhance the open probability of both TASK-3 and TREK-1 channels [19]. However, it is not clear what the consequence of this modulation would be for neuronal excitability as halothane is also known to modulate Na_V_ kinetics and enhance inhibition due to enhancement of GABA_A_ receptor openings. The use of the conductance simulation technique in combination with a recombinant expression system has allowed us to examine the consequence of halothane’s modulation of neuronal excitability in a system where the only possible site of drug action is the K_2P_ channel. As expected, the halothane-induced enhancement of *G*_K_ increased the current required to reach AP threshold with little change in the actual RMP. Therefore, in a neuronal population where the predominant leak conductance arises from a K_2P_ channel population, dampening of neuronal excitability by halothane will predominantly arise from a resistive shunt of any synaptic conductance. Another consequence of increased *G*_K_ in a system like this is the faster membrane time constant that inevitably leads to a briefer AP. The functional significance of any halothane-induced alteration in AP duration is less clear. AP duration is known to influence the transmitter profile in the synaptic cleft by altering the profile of calcium influx into the nerve terminal [4, 46]. A study using a synaptosome preparation has indicated that the amount of glutamate and GABA released from cerebrocortical nerve terminals is influenced by the presence of TREK-1 channels [51], but it remains to be seen what contribution, if any, K_2P_ channels make to the excitability of the intact axon.

If K_2P_ channels were found to contribute to the excitability of the axonal membrane, this could also have important implications for the action of local anaesthetics [30]. A voltage-independent so-called flicker potassium channel has been reported in myelinated axons [32]. This channel population is relatively TEA-insensitive and also blocked by the local anaesthetic bupivacaine [8, 32], leading to the suggestion that K_2P_ channel expression could underlie the potassium permeability of the axonal membrane [35]. Furthermore, the blocking action of bupivicaine is pH-sensitive, suggesting that members of the TASK channel family could underlie this potassium leak conductance. The reduced *G*_K_ resulting from K_2P_ channel block could lead to inactivation of Na_V_ channels due to increased depolarisation block, and this may represent a novel mode of action for local anaesthetics [30]. K_2P_ channels are expressed in dorsal root ganglion and trigeminal ganglia neurons, and data from KO [2] and genetic studies [16, 34] highlight the importance of K_2P_ channels in pain perception and sensitization (for a review, see [38]). However, the exact subcellular localisation of K_2P_ channels in nociceptors has yet to be determined. Therefore, before the involvement of K_2P_ channels in the mechanism of anaesthetic action can be explored further, more information is required on the anatomical location and functional significance of K_2P_ expression in mammalian axons.
